# Subcortical Microstructural Changes Associated with Multiple Metal Exposure

**DOI:** 10.3390/toxics14100911

**Published:** 2026-10-15

**Authors:** Eun-Young Lee

**Affiliations:** Department of Health Care and Science, Dong-A University, Busan 49315, Republic of Korea; enyoungee@dau.ac.kr

**Keywords:** multiple metal exposures, welding, MRI diffusion metrics, subcortical motor areas, Parkinson’s disease, Bayesian Kernel Machine Regression

## Abstract

Metal exposure has been associated with neurodegenerative disorders including Parkinson’s disease (PD). This study examined microstructural changes in subcortical motor areas associated with chronic metal exposure. Subjects with/without a welding history (metal-exposed subjects; *n* = 23 and controls; *n* = 24) were examined. Brain microstructural differences were assessed by MRI diffusion metrics [mean (MD), axial (AD), radial diffusivity (RD) and fractional anisotropy (FA)] in subcortical motor areas [nigro–basal ganglia–thalamic circuit: substantia nigra (SN), caudate nucleus (CN), putamen, globus pallidus (GP), and thalamus; dentatorubral circuit: dentate nucleus (DN) and red nucleus (RN)]. Metal exposure was estimated by lifetime welding years, whole blood metal levels, and T1 and R2* (1/T2*) metrics (estimates of brain metal accumulation). Compared to controls, metal-exposed subjects displayed higher diffusivities in the GP and thalamus (*p*’s < 0.033) but lower FA values in the putamen, SN, and thalamus (*p*’s < 0.039) without differences in DN and RN (*p*’s > 0.115). Blood metal mixtures predicted higher GP and SN T1 metrics (*p*’s < 0.05). Higher T1 metrics predicted higher GP and TH diffusivities and lower SN and TH FA values (*p*’s < 0.044). Higher blood vanadium levels predicted lower SN FA values (*p* = 0.009). Multiple metal exposure may be associated with microstructural compromises within the nigro–basal ganglia–thalamic circuit, while the dentatorubral circuit remains relatively spared, a pattern resembling that of PD at-risk populations.

## 1. Introduction

Chronic and excessive exposure to metals has been associated with neurodegenerative disorders. Once absorbed into the bodily system, these metals bind to specific transporters and can cross the blood–brain barrier, preferentially accumulate in specific brain regions, affect cellular functions by triggering reactive oxygen species and oxidative stress, and thereby contribute to neurodegenerative processes.

Parkinson’s disease (PD) is the second most frequent progressive neurodegenerative disorder after dementia, affecting about 1% of the population aged 60 and above [1]. Its pathological hallmarks are the accumulation of α-synuclein-positive Lewy bodies and the progressive loss of dopamine neurons in the substantia nigra (SN) with subsequent neurodegeneration in the basal ganglia (BG) [2]. Prominent clinical characteristics include motor symptoms such as resting tremor, rigidity, and bradykinesia. Since PD is irreversible once clinical symptoms manifest, tremendous efforts have been exerted to identify risk factors [3]. Despite the growing number of genetic factors associated with PD, the exact causes remain unknown. Thus, there is increased interest in the role of environmental exposure to toxic elements in PD etiology [4]. For example, exposure to well water, pesticides, herbicides, or various metals [e.g., copper (Cu), iron (Fe), manganese (Mn), and lead (Pb)] has been associated with an increased risk of PD and/or PD-like disorders [5]. Exposure to multiple metals is ubiquitous in ambient air pollution, contaminated land, or occupation-related activities. Previous preclinical or epidemiological studies, however, have overlooked multiple metal exposure and their combined and/or interaction effects to evaluate PD-related brain changes [5].

MRI techniques enable noninvasive in vivo evaluation of neurodegenerative changes and have been utilized to pinpoint changes in brain regions related to PD [6]. Previous studies have reported altered functional or white-matter-based structural connectivity and microstructural changes in several cortical and subcortical gray matter areas, including the SN and BG in early PD or PD-at-risk populations [7,8]. Diffusion-weighted imaging (DWI) techniques that measure the random translational motions of water molecules in tissues are known to sensitively capture microstructural tissue degradation, including compromised neural tissue integrity or axonal demyelination [9]. For instance, PD patients at an early stage had altered diffusion metrics [e.g., lower fractional anisotropy (FA), higher diffusivities and free water, or altered kurtosis metrics], particularly in the SN and BG [10,11], that were associated with dopaminergic neural degeneration [12].

The present study examined the effects of multiple metal exposure via welding fumes on diffusion metrics in subcortical motor areas. Welding fumes are composed of diverse metals, encompassing both essential elements [e.g., Cu, Fe, and Mn] and toxic, non-essential ones (e.g., Pb). Thus, studying the effects of welding-related metal exposure can provide insights into multiple-metal-related neurotoxicity. To estimate the current internal dose of metal exposure, whole-blood metal levels and MRI T1 and R2* metrics were quantified. To estimate the cumulative brain burden of metal exposure, MRI diffusion metrics were computed. The following specific hypotheses were tested: (H1) Subjects with chronic welding exposure (metal-exposed subjects) will have higher MRI T1 and R2* metrics (MRI-based surrogate markers of brain metal content) and altered MRI diffusion metrics (estimates of brain microstructures) in subcortical motor areas compared to controls. (H2) In metal-exposed subjects, blood metal mixtures will predict subcortical MRI metrics. A Bayesian kernel machine regression (BKMR) analysis [13] was performed to flexibly model the individual, combined, and interaction effects of multiple blood metals on subcortical MRI metrics. This study also sought to identify individual metals that may be responsible for subcortical MRI metrics using multiple linear/polynomial regression and BKMR analysis.

## 2. Materials and Methods

### 2.1. Study Subjects

Forty-seven subjects (23 and 24 with/without a history of welding) comprised the final sample. Metal-exposed subjects were volunteers with a minimum of 10 years of welding experience in shipyard companies in Busan, South Korea, or nearby areas. Controls had various occupations with no history of welding and were enrolled from local communities in Busan, South Korea, or nearby areas. None of the subjects had a history of any neurological disorders. The Unified PD Rating Scale-motor-III (UPDRS-III) scores of all participants were ≤5.

### 2.2. Statements and Declarations

This study adhered to the principles outlined in the Declaration of Helsinki, and all participants provided written informed consent under protocols approved by the Dong-A University Institutional Review Board.

### 2.3. Occupational Metal Exposure History and Whole-Blood Metal Levels

#### 2.3.1. Occupational Metal Exposure History

Exposure to metals was estimated using metal exposure questionnaires that measured lifetime welding years (YrsW).

#### 2.3.2. Whole-Blood Metal Levels

To estimate the current internal dose of metal exposure, whole-blood levels of aluminum (Al), Cu, Fe, Mn, Pb, and vanadium (V) were measured and analyzed by Inductively Coupled Plasma Mass Spectrometry (ICP-MS; Agilent 7700× ICP-MS, Agilent Technologies, Santa Clara, CA, USA) at Dong-A University Hospital. Whole-blood samples were collected on the day of the MRI scan (Siemens, Magnetom Vida, Erlangen, Germany) and stored at −70 °C until analysis. The ICP-MS instrument was fitted with a concentric glass nebulizer and a Peltier-cooled glass cyclonic spray chamber. The dilution solution was prepared using 2% 1-butanol, 0.05% EDTA, 0.05% Triton X-100, and 1% NH4OH (Sigma-Aldrich, St. Louis, MO, USA). Multi-element solutions (10 mg/L, Agilent Technologies, Santa Clara, CA, USA) were used as calibration standards. Rhodium (10 mg/L, Agilent Technologies) was used as an internal standard. To validate the analytical method, Clinchek Whole Blood Controls Level I and II (RECIPE CHEMICALS, Munich, Germany) were used as matrix standards [14].

### 2.4. MRI Image Acquisition and Image Processing

#### 2.4.1. MRI Image Acquisition

All MR images were acquired using a Siemens 3T scanner (Siemens, Magnetom Vida, Erlangen, Germany) with a 64-channel head coil. First, high-resolution T1-weighted (T1W) scans were collected to enable anatomical segmentation. For T1W, MPRAGE sequences with Repetition Time (TR)/Echo Time (TE) = 1540/2.3 ms, flip angle = 9°, FoV/matrix = 512 × 512 mm, slice number = 176 (with no gap), and voxel spacing 0.5 × 0.5 × 1 mm were used. For R1 (1/T1), a fast T1 mapping sequence was obtained with TR/TE = 15/1.45 ms, flip angles = 4/25, FoV/matrix = 160 × 160 mm, slice number = 192, and voxel spacing = 1.56 × 1.56 × 1 mm. For R2* (1/T2*), multi-gradient-echo susceptibility-weighted images were obtained using eight echoes: TR/TE = 55/6.2~49.6 ms, flip angle = 15°, FOV/matrix = 256 × 256 mm, slice number = 80, and voxel spacing = 1 × 1 × 2 mm. For DWI, TR/TE = 4700/93, flip angle = 90°, FoV/matrix = 128 × 128 mm, voxel spacing = 2 × 2 × 2 mm, slice number = 72, b = 0, 600, 1200, and 2000, and number of directions for each nonzero b-value = 30.

#### 2.4.2. Defining Brain Regions of Interest

**Figure 1:**
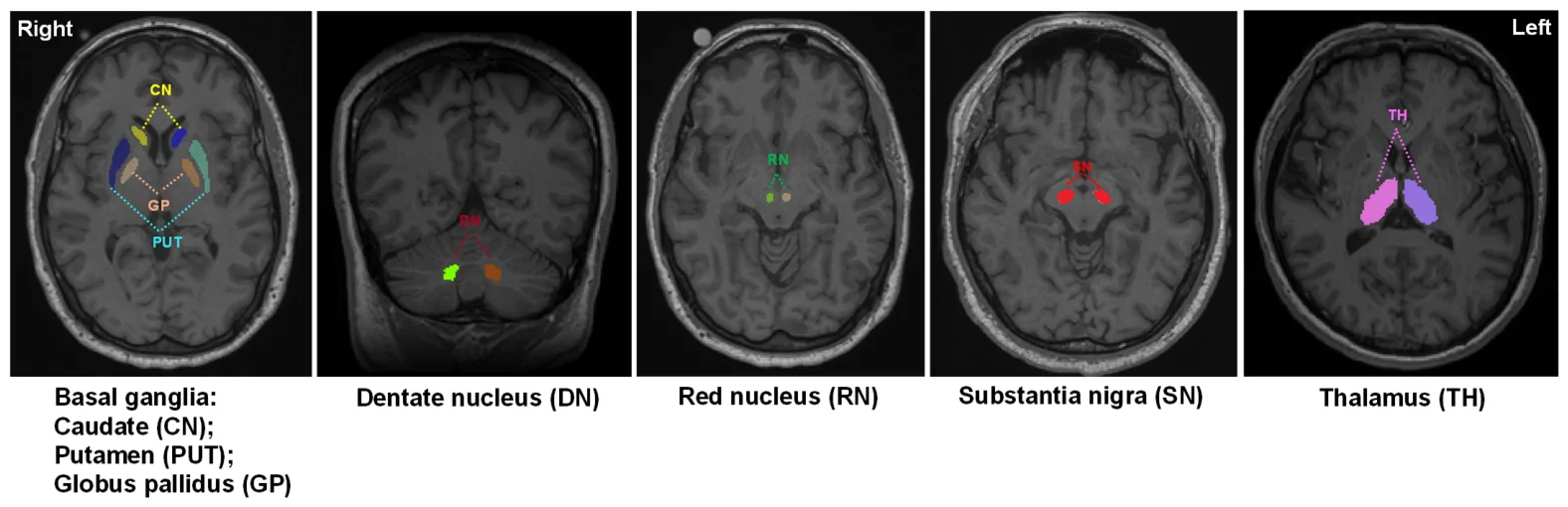
Regions of interest [caudate nucleus (CN), dentate nucleus (DN), globus pallidus (GP), putamen, red nucleus (RN), substantia nigra (SN), and thalamus (TH)].

Bilateral motor-related subcortical areas [caudate nucleus (CN), dentate nucleus (DN), globus pallidus (GP), putamen, red nucleus (RN), substantia nigra (SN), and thalamus (TH)] were selected as regions of interest (ROIs) (Figure 1). ROIs were delineated on the T1W images using automatic atlas-based parcellation software (AutoSeg v3.0). The segmentation outputs underwent visual quality control by a rater blinded to group allocation. MRI metrics were computed on a voxel-wise basis and subsequently averaged across the entire ROI.

#### 2.4.3. Brain MRI Measurements

***T1W intensity****:* T1W scans were skull-stripped and bias-field-corrected to control for intensity variations caused by imperfect magnetic fields. Histogram-matching intensity standardization was subsequently implemented to minimize magnetic field inhomogeneity across subjects.

***MRI R1 values:*** To calculate R1 values, whole-brain T1 scans were coregistered onto the T1W reference images via affine transformation using 3D Slicer.

MRI R2* *values*: The magnitude images of multigradient-echo images were used to generate R2* maps by applying a voxel-wise linear least-squares fit to a monoexponential decay function. The generated R2* maps were coregistered onto the T1W reference images via affine transformation using 3D Slicer.

***Diffusion* *metrics:*** DWI data underwent quality assurance using the FSL diffusion toolbox that checks diffusion images for appropriate quality by calculating the inter-slice and intra-class correlation within images, and then corrects for distortions induced by eddy currents and head motion. Diffusion tensor imaging (DTI) maps were then estimated using weighted least squares and coregistered onto T1W scans using ANTS. Four DTI [mean (MD), axial (AD), radial diffusivity (RD), and fractional anisotropy (FA)] metrics were calculated based on three diffusivity eigenvalues (λ1, λ2, λ3). FA quantifies the degree of directional diffusion, calculated from the normalized pairwise differences across the diffusion tensor’s three eigenvalues. AD is defined as the principal eigenvalue (λ1), whereas RD corresponds to the average of the remaining perpendicular eigenvalues (λ2 and λ3). MD is calculated as the average across all three eigenvalues, quantifying the overall magnitude of water diffusion.

### 2.5. Statistical Analysis

Group comparisons for demographic data were performed using independent two-sample *t*-tests or a χ^2^ test. For group comparisons of whole-blood metal and MRI metrics, multivariate analysis of covariance (MANCOVA) was performed to account for intercorrelations among dependent variables while adjusting for age. Bilateral MRI metrics were averaged within each subject. Whole-blood metal concentrations were log-transformed to mitigate the influence of extreme values.

Multiple linear or polynomial regression analysis was performed to examine the relationship between metal exposure dose and response (MRI T1, R2*, and diffusion metrics) with adjustment for age in metal-exposed subjects and controls, respectively. MRI T1 and R2* metrics were plotted against occupational metal exposure history and blood metal metrics; the diffusion metrics were plotted against metal exposure measures [occupational metal exposure history; blood metal levels; MRI T1 and R2*] using Loess plots. Depending on the pattern of the associations observed in the plots, linear or higher-order polynomial regression models were tested. Subsequently, a sensitivity analysis for the regression analysis was performed with adjustments for age and smoking status, to test whether the dose–response relationship would significantly be modified by the smoking status. The Bayesian kernel machine regression (BKMR) analysis [13] flexibly models non-linear and non-additive joint effects between multiple blood metal and MRI metrics (T1, R2*, and diffusion metrics) using a Markov Chain Monte Carlo (MCMC) algorithm. This analysis method allows testing of the combined effects of multiple blood metals and their pairwise interactions alongside independent individual metal effects. Statistical significance was set at α = 0.05. Individual group comparison results were adjusted for multiple comparisons using the Stepdown Bonferroni procedure to maintain the familywise error rate (FWER) at *p* = 0.05. Uncorrected raw *p*-values were reported but significant results with FWER correction were indicated. SAS 9.4 and R 4.3.2 were employed for statistical analysis.

## 3. Results

### 3.1. Demographics and Metal Exposure Characteristics

Metal-exposed subjects were younger than controls (*p* = 0.036). There were no significant group differences in the frequency of smokers, alcohol usage, or UPDRS-III scores (*p’s* > 0.238). Metal-exposed subjects displayed higher occupational exposure history (*p* < 0.001) but no differences in any whole-blood metal levels of Al, Cu, Fe, Mn, Pb, and V (*p’s* > 0.194; Table 1).

### 3.2. Group Comparisons of MRI TI and R2* Metrics

**Figure 2:**
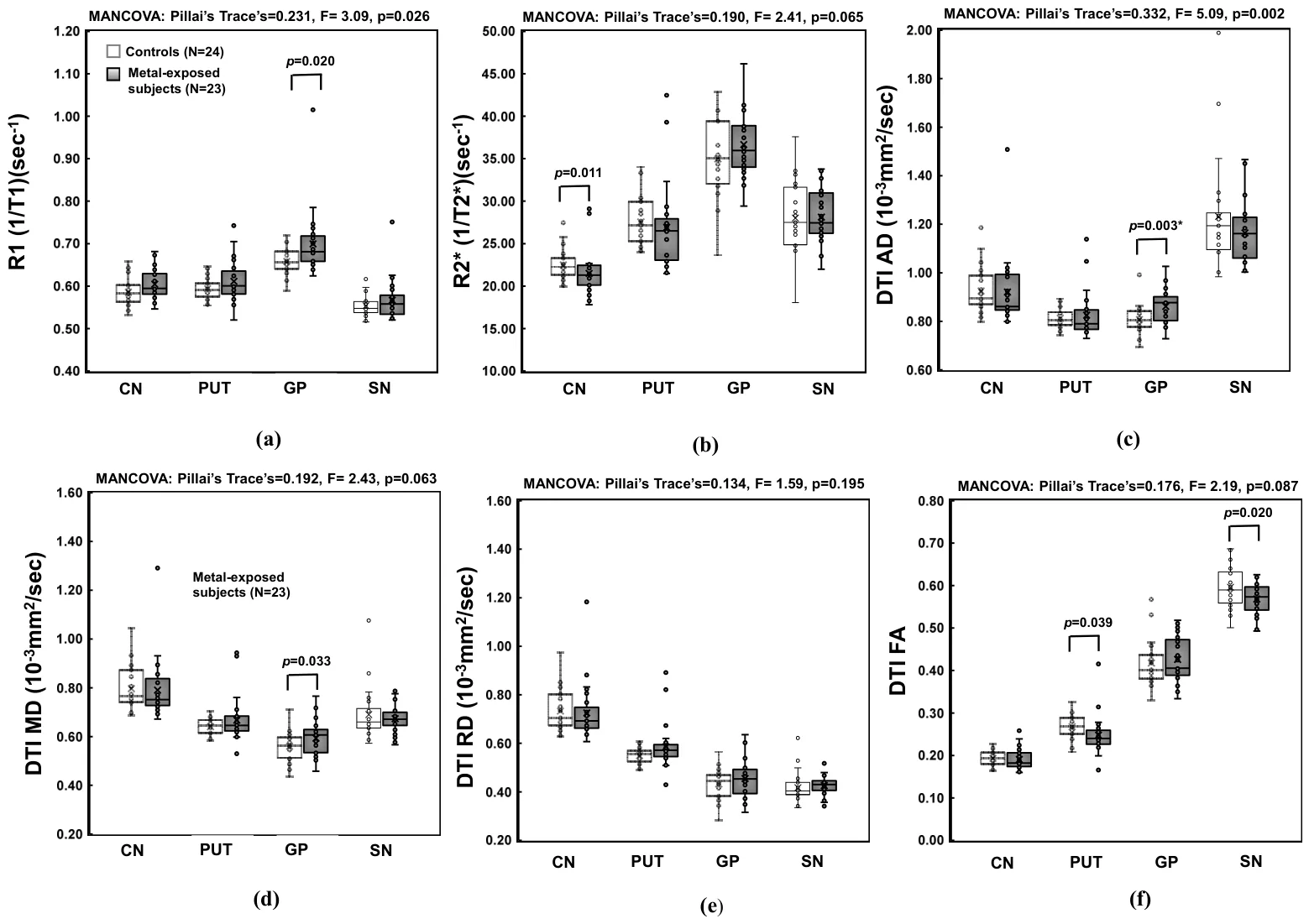
Boxplots indicating MRI metrics (y-axis): (**a**) R1 (1/T1), (**b**) R2*, (**c**–**f**) diffusion tensor imaging (DTI) metrics [(**c**) axial (AD), (**d**) mean (MD), (**e**) radial diffusivity (RD), and (**f**) fractional anisotropy (FA)] in regions of interest [x-axis; caudate nucleus (CN), dentate nucleus (DN), globus pallidus (GP), putamen, red nucleus (RN), substantia nigra (SN), and thalamus (TH)]: for metal-exposed subjects and controls; The small circles indicate outliers falling beyond the 1.5 × interquartile range; * indicates significance (*p* < 0.05) after correction for multiple group comparisons using the Stepdown Bonferroni method.

There were significantly higher R1 values for metal-exposed subjects throughout all ROIs (Pillai’s Trace = 0.295, F = 2.27, *p* = 0.049) but no overall group differences in R2* or T1W metrics (*p*’s > 0.173). Individual comparisons revealed that metal-exposed subjects had higher GP R1 (*p* = 0.020) but lower CN R2* values (*p* = 0.011; see Figure 2a,b).

### 3.3. Group Comparisons of Diffusion Metrics

Compared to controls, metal-exposed subjects demonstrated higher DTI AD and MD values throughout all ROIs (Pillai’s Trace = 0.356, F = 3.00, *p* = 0.013 for AD and Pillai’s Trace = 0.319, F = 2.55, *p* = 0.030 for MD; Figure 2c,d) but not higher RD or FA values (*p*’s > 0.183; Figure 2e,f). Individual comparisons revealed higher AD and MD values in the GP (*p* = 0.003 and *p* = 0.033, respectively) and higher MD and RD values in the TH (*p* = 0.028 and *p* = 0.015, respectively), but lower FA values in the PUT, SN, and TH (*p*’s < 0.039) for metal-exposed subjects. Higher GP AD values remained significant after FWER-correction. There were no significant group differences in the CN, DN, or RN (*p*’s > 0.115).

### 3.4. Associations of Occupational Metal Exposure History and Blood Metals with MRI T1 and R2* Metrics

#### 3.4.1. Occupational Metal Exposure History

Lifetime welding years did not predict the MRI T1 or R2* metrics of any ROI (*p*’s > 0.074).

#### 3.4.2. Blood Metal Levels

For metal-exposed subjects, higher blood V levels linearly predicted higher R1 values in the GP, SN, and TH (β’s > 0.060, t’s > 2.22, *p*’s < 0.042; Table 2a). Higher blood Mn levels linearly predicted higher TH R1 values (ß = 0.039, t = 2.55, *p* = 0.022). Higher blood Pb levels nonlinearly predicted higher R1 values in the GP and SN: R1 values started to increase above a certain threshold of blood Pb levels (ß’s > 0.101, t’s > 5.35, *p*’s < 0.001). Higher blood Mn and V but lower blood Fe levels linearly predicted higher T1W values in the PUT, GP, SN, and TH (ß’s > 39.392, t’s > 2.18, *p*’s < 0.045 for Mn; ß’s > 122.287, t’s > 4.53, *p*’s < 0.001 for V; ß’s < −134.888, t’s < −2.52, *p*’s < 0.024 for Fe). Higher blood V but lower blood Fe levels linearly predicted higher T1W values in the CN, DN, and RN (ß’s > 120.863, t’s > 3.91, *p*’s < 0.001 for V and ß’s < −126.130, t’s < −2.45, *p*’s < 0.027 for Fe; Table 2b). Higher blood Cu levels linearly predicted higher R2* values in the PUT, GP, RN, SN, and TH (ß’s > 7.252, t’s > 2.18, *p*’s < 0.046; Table 2c). For controls, none of the blood metals significantly predicted the T1 or R2* metrics of any ROI (*p*’s > 0.106). A sensitivity analysis with adjustments for age and smoking status revealed similar dose (occupational metal exposure history and blood metals)–response (MRI T1 and R2*) associations (Supplementary Table S1a–c).

#### 3.4.3. BKMR Analysis of Blood Metal Mixture Effects on T1 and R2* Metrics

**Figure 3:**
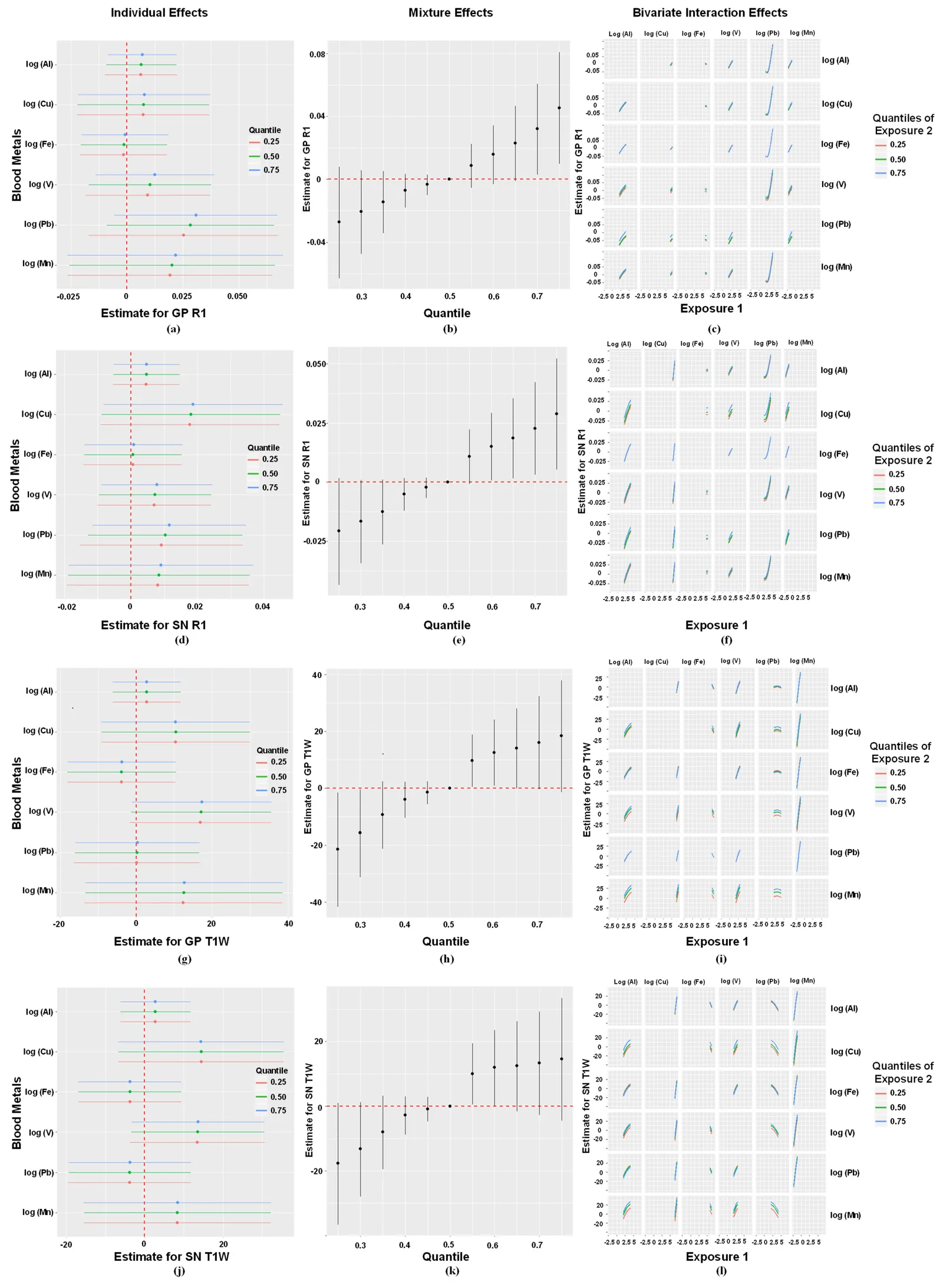
BKMR (Bayesian Kernel Machine Regression) analysis results showing the effects of individual metals at quantiles of 0.25, 0.50, and 0.75 on R1(1/T1) values in the (**a**) globus pallidus (GP) and (**b**) substantia nigra (SN) and T1-weighted (T1W) intensities in the (**c**) GP and (**d**) SN; overall metal mixture at various quantiles on R1 values of the (**e**) GP and (**f**) SN and T1W intensities in the (**g**) GP and (**h**) SN; and bivariate exposure–response functions of a single exposure variable (exposure 1) in the (**i**) GP and (**j**) SN and T1W intensities in the (**k**) GP and (**l**) SN when the second exposure variable (exposure 2) is fixed at quantiles of 0.25, 0.50, and 0.75 and the remaining exposure variables fixed to the median. The red dotted lines indicate the zero point of the parameter estimates for the dependent variables.

Within metal-exposed subjects, no individual blood metal variable was a significant predictor of T1W or R1 values in any ROI. Instead, there were significant overall metal mixture effects on higher R1 at quantiles >0.70 and T1W at quantiles between 0.5 and 0.6 in the GP and SN (Figure 3). There were no significant interactions between any pair of blood metals in the mixture. For R2* metrics, there were no significant individual, mixture, or interaction effects of multiple blood metals on the R2* values of any ROI (*p*’s > 0.05). For controls, there were no significant individual, mixture, or interaction effects of multiple blood metals on the MRI T1 or R2* metrics of any ROI (*p*’s > 0.05).

### 3.5. Associations of Occupational Metal Exposure History and Blood Metals with Diffusion Metrics

#### 3.5.1. Occupational Metal Exposure History

**Figure 4:**
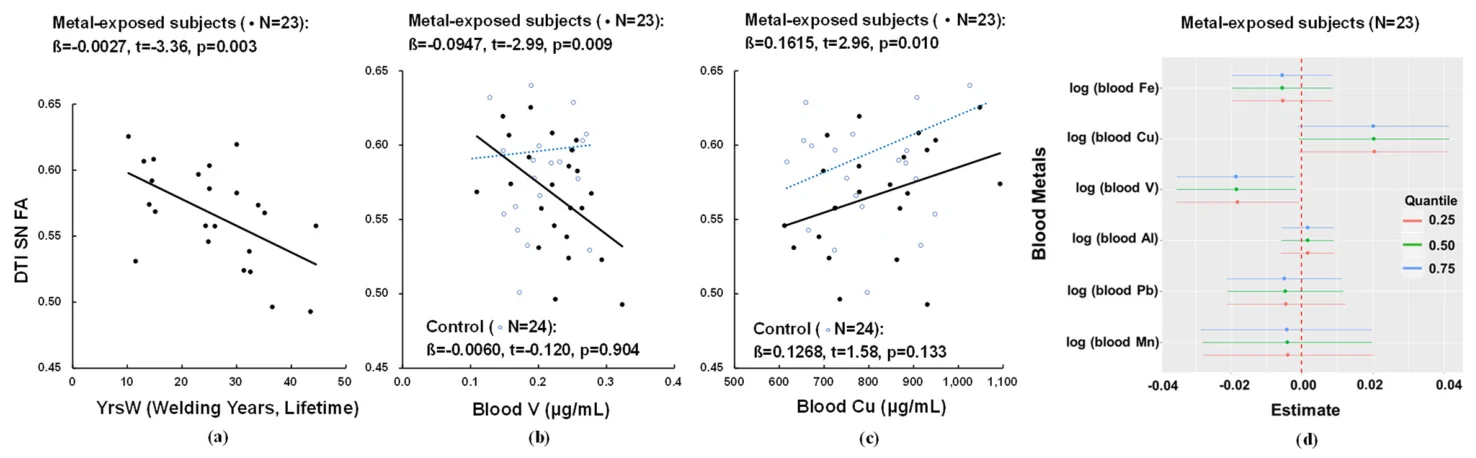
Scatter plots with regression lines showing diffusion tensor imaging (DTI) fractional anisotropy (FA) values in the substantia nigra (SN) (DTI SN FA; y-axis) vs. (**a**) lifetime years welding (YrsW; x-axis) for metal-exposed subjects; (**b**) whole blood vanadium (V) levels (Blood V; x-axis) and (**c**) whole blood copper (Cu) levels (Blood Cu; x-axis) for both metal-exposed subjects and controls; and (**d**) BKMR (Bayesian Kernel Machine Regression) analysis results showing the effects of individual metals at quantiles of 0.25, 0.50, and 0.75 for metal-exposed subjects. The red dotted lines indicate the zero point of the parameter estimate for the dependent variable of DTI SN FA.

For metal-exposed subjects, higher lifetime welding years linearly predicted lower SN FA values (ß = −0.0027, t = −3.36, *p* = 0.003; Figure 4a and Table 2d).

#### 3.5.2. Blood Metal Levels

For metal-exposed subjects, higher blood V but lower Cu levels linearly predicted lower SN FA values [ß = −0.0947, t = −2.99, *p* = 0.009 for V and ß = 0.1615, t = 2.96, *p* = 0.010 for Cu; Figure 4b,c]. Polynomial regression analysis revealed that PUT FA values had a significant second-order relationship with blood Cu levels (β = 0.9607, t = 2.87, *p* = 0.012; Supplementary Figure S1a): PUT FA values exhibited a significant increase once blood Cu levels exceeded a specific threshold. There was no significant linear or polynomial relationship between the remaining blood metal levels and other diffusion metrics. For controls, none of the blood metal concentrations significantly predicted diffusion metrics (*p*’s > 0.226).

#### 3.5.3. BKMR Analysis of Blood Metal Mixture Effects on Diffusion Metrics

For metal-exposed subjects, elevated blood V levels were the only significant predictor of lower SN FA values (*p* < 0.05; Figure 4d). None of the remaining individual blood metal variables was a significant predictor of diffusion metrics of any ROIs. There were also no significant overall metal mixture or interaction effects of multiple blood metals on the diffusion metrics of any ROIs (Supplementary Figure S1c,d). For controls, there were no significant individual, mixture, or interaction effects of multiple blood metals on the diffusion metrics of any ROIs (*p*’s > 0.05).

#### 3.5.4. MRI-Based Surrogate Markers of Brain Metal Content (MRI T1 and R2*)

For metal-exposed subjects, polynomial regression analysis showed that diffusivity values had a significant second-order relationship with T1W or R1 values in the GP and TH (β’s > 0.15 × 10^−4^, t’s > 2.46, *p*’s < 0.024; Table 2d): the diffusivity values increased once R1 or T1W values exceeded specific threshold values. The FA values in the SN and TH, on the other hand, exhibited a significant decrease once R1 values exceeded a specific threshold (β’s < −3.9598, t < −2.16, *p*’s < 0.044). PUT FA values exhibited a significant increase once PUT R2* values exceeded a specific threshold (β = 3.74 × 10^−3^, t = 3.13, *p* = 0.006; Supplementary Figure S1b). For controls, higher PUT R2* values linearly predicted higher PUT FA values (β = 0.005, t = 3.39, *p* = 0.003). None of the remaining T1 or R2* metrics significantly predicted the diffusion metrics of any ROI (*p*’s > 0.113). A sensitivity analysis with adjustments for age and smoking status revealed similar dose (occupational metal exposure history, blood metal, and MRI T1 and R2* metrics)–response (MRI diffusion metrics) associations (Supplementary Table S1d).

## 4. Discussion

The present study examined microstructural changes in subcortical motor areas associated with multiple metal exposure. First, metal-exposed subjects demonstrated higher R1 but lower R2* metrics in the BG, suggestive of altered brain metal accumulation (e.g., Mn and Fe, respectively). Second, they had altered diffusion metrics in the BG, SN, and TH with no differences in the DN and RN. Third, blood metal mixtures predicted T1 metrics in the GP and SN. Fourth, higher subcortical T1 metrics predicted higher GP and TH diffusivities but lower SN and TH FA values. In addition, a greater long-term occupational metal exposure history and elevated blood V levels predicted lower SN FA. These findings suggest that multiple metal exposure may be associated with microstructural compromises of the nigro–basal ganglia–thalamic circuit, while the dentatorubral circuit was relatively spared, a pattern resembling that of PD at-risk populations.

### 4.1. Altered MRI T1 and R2* Metrics in the BG

Our metal-exposed subjects showed higher R1 values throughout all ROIs, and that was particularly true in the GP. T1 metrics (R1 and T1W values) are known to sensitively reflect brain metal concentrations such as Mn due to its paramagnetic nature [15]. The BKMR analysis further suggested that blood metal mixtures may lead to higher subcortical R1 values, particularly in the GP and SN.

Our metal-exposed subjects also showed lower R2* values in the CN, a metric known to sensitively reflect brain Fe accumulation [16], suggestive of lower brain Fe content compared to controls. This may partly be due to the toxicokinetics of Fe and Mn, particularly their competition for shared transport mechanisms to enter the brain [17]: Higher Mn brain accumulation, evidenced by higher R1 values, may have hindered Fe brain entry. The different spatial dynamics of R1 and R2* metrics suggest that different metals may preferentially accumulate in different brain regions. It is intriguing to note that higher blood Cu, not Fe, levels predicted higher R2* values in the SN and BG. Cu is known to interact with the molecular pathways governing Fe metabolism [18]: Excessive peripheral Cu levels can lead to brain Fe overload by interfering with Fe metabolism or as an inflammatory response to higher Cu. For example, higher BG R2* values were observed in deceased patients with Wilson’s disease, which were associated with brain Fe but not with brain Cu levels [19].

### 4.2. Altered Diffusion Metrics in the Nigro–Basal Ganglia–Thalamic Circuit for Metal-Exposed Subjects

Our metal-exposed subjects had altered diffusion metrics in the SN, BG (PUT and GP), and TH. Dopaminergic neurons are projected from the SN to the striatum (CN and PUT) of the BG via the nigrostriatal pathway. Once entering the BG, neuronal signals are transmitted to the (internal) GP, a primary output nucleus, via the direct or indirect BG pathway and continue through the BG–thalamic–cortical circuit to facilitate desired movements while suppressing competing, unwanted ones [20]. Although the exact nature of the diffusion metrics is unclear, they may be sensitive in capturing neuropathological processes (e.g., reduced tissue integrity or demyelination) at microstructural levels [9,10], suggesting compromised microstructures of the nigro–BG–thalamic circuit in metal-exposed subjects. These results are consistent with features observed in early PD or PD at-risk populations. Note that the nigro–BG–thalamic–cortical circuit is known to be dysfunctional in PD due to dopaminergic neuronal deaths in the SN and subsequent neurodegeneration in the BG [21]. Prior studies have reported altered diffusion metrics in the nigro–BG–thalamic circuit, particularly in the SN (e.g., lower SN FA) in early PD or PD at-risk populations [8,22]. Lower SN FA values in PD were associated with reduced dopamine transporter availability [12]. Lower SN FA values were also observed in PD at-risk populations [23]. Nevertheless, caution needs to be taken in interpreting the current findings because diffusion metrics are not considered a diagnostic marker for PD. Further studies are warranted to better delineate the associations between altered subcortical diffusion metrics and PD-related biomarkers (e.g., neuromelanin-sensitive or dopamine transporter imaging) in metal-exposed subjects.

It is worthwhile to note that relatively preserved diffusion metrics were observed in the DN and RN. The DN is the main output nucleus of the cerebellum, projected to the RN via the superior cerebellar peduncle, playing a key role in the planning and timing of externally guided movements [24]. The involvement of this dentatorubral circuit in PD pathology is under debate. The DN and BG are interconnected via the TH [25]. Correspondingly, lower volumes and functional connectivity of the DN with other brain areas have been reported even in early PD [26,27]. Other studies, however, suggested that the dentatorubral circuit may be relatively preserved or even overactivated in early PD or in PD at-risk populations to compensate for the compromised nigro–BG–thalamic–cortical circuit [21]. The current findings support relatively preserved dentatorubral circuits despite compromised nigro–BG–thalamic circuits in metal-exposed subjects.

### 4.3. MRI T1 Metrics May Predict Altered Diffusion Metrics in the Nigro–Basal Ganglia–Thalamic Circuit

In our metal-exposed subjects, higher T1 metrics in the GP, TH, and SN predicted higher GP and TH diffusivities but lower FA values in the SN and TH. Coupled with the multiple regression and BKMR analysis, the T1-diffusion metric associations suggest that altered diffusion may partly be due to the accumulation of metal mixtures in the brain. In addition, our metal-exposed subjects showed lower PUT FA, which was associated with lower BG R2* values, suggesting diminished microstructural tissue organization in the PUT when brain Fe levels get lower (than an optimal level) due to competing dynamics between metals (e.g., Fe and Mn) for brain entry [17]. Together, the present findings suggest that multiple brain metals (e.g., Fe, Mn, or V) estimated by MRI-based surrogate markers individually or as mixtures may contribute to microstructural changes in different regions of the nigro–BG–thalamic circuit. Future studies with larger samples are warranted to test the causal paths linking exposure to metal mixtures to cumulative brain burden, depending on the toxicokinetics of individual metals.

### 4.4. Chronic V Exposure May Predict Altered Diffusion Metrics in the SN

Across different statistical models of multiple linear/polynomial regression and BKMR analysis, blood V levels were the only significant factor predicting altered diffusion metrics in the SN (e.g., lower SN FA values) for our metal-exposed subjects. Coupled with the inverse relationship between SN FA and lifetime welding years, lower SN FA values may be exacerbated with higher and more prolonged V exposure.

V is an essential metal for normal cellular function, but excessive V exposure can be neurotoxic [28,29]. V is present in the environment through natural sources (e.g., volcanoes, forest fires, or fossil fuels), and is also widely used in industry, such as welding processes for shipyards and steel or battery production, due to its capacity to enhance steel hardness and ductility. Significantly higher blood V levels were reported in welders after their shift compared to before, or compared to unexposed controls, whereas blood V levels in unexposed controls were often below the limit of detection (e.g., around 0.01 to 0.05 μg/L) [30,31], rendering V exposure a relevant factor in the neurotoxicity associated with welding-related multiple metal exposures. Workers occupationally exposed to V also demonstrated neurobehavioral dysfunctions (e.g., lower psychomotor speed, spatial cognition, and memory performance) or psychiatric problems such as emotional irritation, anger, and depression [29]. V can enter the brain by binding to transporters (e.g., transferrin), accumulate in the olfactory bulb, brain stem, and cerebellum, and may increase reactive oxygen species that lead to oxidative stress and subsequent cell death [28]. Chronic excessive V exposure has been associated with increased risk of neurodegenerative disorders such as PD: V-exposed animals showed dopaminergic neural degeneration in the olfactory bulb and SN, regions particularly affected in early PD. Moreover, increased α-synuclein expression levels in the SN were observed in animals exposed to V or other metals (e.g., Mn). Interestingly, the magnitude of the increased α-synuclein expression levels was greater during exposure to V than to Mn, stressing the importance of considering V exposure in assessing metal-related PD risk [32]. The current finding indicates that chronic V exposure may be associated with lower SN FA, a feature often observed in early PD or PD-at-risk populations. This is the first study demonstrating the dose (e.g., V exposure)–response (microstructural alterations in the SN) relationship in human subjects occupationally exposed to V. It is worthwhile to note that there were no significant group differences in blood V levels, although our metal-exposed subjects had comparable whole blood V levels to those in other studies with exposed subjects [31,33], suggesting that our unexposed controls might have had transient and irregular sources of V exposure. The finding of significant blood V-MRI associations only in occupationally exposed subjects highlights the importance of chronic metal exposure in long-term brain burden.

## 5. Conclusions and Limitations

The current study demonstrated that multiple metal exposure may affect microstructures in the nigro–BG–thalamic circuit, the neuroanatomical loci of which are affected in PD. The robust dose–response relationship between V exposure and SN FA values in human subjects occupationally exposed to multiple metals is novel. This underscores the importance of examining multiple metals, including V, in evaluating neurotoxic processes and increased risks of neurodegenerative disorders such as PD in subjects occupationally exposed to multiple metals.

The current study has several limitations: First, one-time cross-sectional whole-blood metal measurements were performed to assess the peripheral internal dose of metal exposure. Blood metal metrics reflect short-term exposure dynamics only up to a few weeks and are not sufficiently sensitive to capture cumulative exposure effects. Future studies with multiple measurements of blood metals or with cumulative metal exposure metrics (e.g., bone and nails) are warranted to better elucidate the effects of chronic metal exposure on cumulative brain burden. Second, caution needs to be taken in interpreting the current findings because the sample sizes were relatively small and factors such as the presence of genetic disorders or differences in cellular transport, chaperone, and storage systems can alter brain metal accumulation, which, in turn, may increase long-term brain burden, as reflected by the altered diffusion metrics observed in the current study. Finally, welding fumes contain a complex aerosol containing metal oxides, ultrafine particles, and toxic components other than those measured in the present study, depending on the welding process that may have contributed to the current findings. Future studies are warranted to address the dynamics of various co-exposures, genetic predispositions, and the networks of chaperones and transport proteins to delineate the causal links between chronic metal exposure and long-term brain burden.

## Figures and Tables

**Table 1:** Summary demographic data for metal-exposed subjects and controls.

	Controls (*N* = 24) Mean ± SD	Metal-Exposed Subjects (*N* = 23) Mean ± SD	*p*-Values
Age (years)	60.7 ± 4.1	58.2 ± 3.8	0.036
Gender (frequency; m/f)	15/9	18/5	0.238
Smoking status (frequency; yes/no)	14/10	16/7	0.547
Alcohol (frequency; yes/no)	14/10	21/2	0.245
BMI	23.5 ± 2.7	23.2 ± 2.6	0.688
UPDRS-III	1.6 ± 1.4	2 ± 1.6	0.459
***Occupational metal exposure history***			
YrsW (years)	0	25.7 ± 10.0	<0.001
***Whole-blood metal levels* MANOVA: Pillai’s Trace’s = 0. 119, F = 0.90, *p* = 0.504**			
Al (μg/L)	21.6 ± 3.9	21.1 ± 9.4	0.368
Cu (μg/L)	813.4 ± 120.1	816.3 ± 126.9	0.952
Fe (mg/L)	459.8 ± 75.4	485.1 ± 67.9	0.225
Mn (μg/L)	12.3 ± 4.2	13.0 ± 4.1	0.585
Pb (μg/L)	15.1 ± 4.8	18.9 ± 11.6	0.194
V (μg/L)	0.21 ± 0.05	0.22 ± 0.05	0.216

Data represents the mean ± SD or frequency. Groups were compared using two-sample *t*-tests or a χ^2^ test. Metal exposure measures were compared using multivariate analysis of variance (MANOVA). Abbreviations: BMI = Body Mass Index; UPDRS = Unified PD Rating Scale; YrsW = lifetime welding years.

**Table 2:** Linear or polynomial regression analysis evaluating the effects of metal exposure measures on MRI metrics in metal-exposed subjects.

*Outcome Variables*	*Selected Predictors*	*Parameter Estimates (ß)*	*t-Value*	*p-Value*
*Metal-exposed subjects (N = 23)*				
***a. R1 metrics***				
***Linear***				
GP	V	0.186	2.65	0.018
SN		0.103	2.22	0.042
TH		0.060	2.61	0.020
	Mn	0.039	2.55	0.022
***Polynomial***				
GP	Pb	0.137	5.35	<0.001 *
SN		0.101	8.51	<0.001 *
***b. T1W metrics***				
***Linear***				
CN	Fe	−126.130	−2.52	0.024 *
	V	120.863	4.15	<0.001 *
PUT	Fe	−155.020	−3.09	0.008 *
	Mn	43.438	2.21	0.043
	V	143.387	4.90	<0.001 *
GP	Fe	−162.828	−2.86	0.012 *
	Mn	56.144	2.51	0.024 *
	V	162.677	4.90	<0.001 *
DN	Fe	−142.532	−2.45	0.027
	V	143.989	4.26	<0.001 *
RN	Fe	−143.226	−2.66	0.018 *
	V	122.740	3.91	0.001 *
SN	Fe	−151.599	−2.91	0.011 *
	Mn	45.736	2.25	0.040
	V	137.080	4.53	<0.001 *
TH	Fe	−134.888	−2.93	0.010 *
	Mn	39.392	2.18	0.045
	V	122.287	4.56	<0.001 *
c. R2* metrics				
***Linear***				
PUT	Cu	23.660	2.56	0.022 *
GP		20.276	2.69	0.018 *
RN		17.446	2.44	0.028
SN		16.841	2.72	0.016 *
TH		7.252	2.18	0.046
***d. Diffusion metrics***				
***Linear***				
SN FA	YrsW	−0.0027	−3.36	0.003 *
	Cu	0.1615	2.96	0.010 *
	V	−0.0947	−2.99	0.009 *
***Polynomial***				
PUT FA	Cu	0.961	2.87	0.012 *
	PUT R2*	3.74 × 10^−4^	3.13	0.006 *
GP AD	GP T1W	0.15 × 10^−5^	2.57	0.019 *
MD		0.15 × 10^−5^	2.46	0.024 *
SN FA	SN R1	−3.9598	−2.16	0.044
TH FA	TH R1	−29.447	−4.15	<0.001 *
MD		49.838	3.41	0.003 *
RD		59.386	4.08	<0.001 *

Data represents parameter estimates (ß) and corresponding *t*- and *p*-values of (a–c) exposure measures (lifetime welding years and blood metals) to predict MRI T1 and R2* metrics (MRI-based surrogate markers of brain metal content) and (d) metal exposure measures (lifetime welding years, blood metals, and MRI T1 and R2* metrics) to predict diffusion metrics (estimate of long-term brain burden). The analysis was adjusted for age. * indicates significant results after Stepdown Bonferroni multiple comparison correction of *p* < 0.05.

## Data Availability

The data presented in this study are available on request from the corresponding author due to privacy and ethical restrictions.

## Supplementary Materials

The following supporting information can be downloaded at: https://www.mdpi.com/article/10.3390/toxics14100911/s1, Supplementary Figure S1: Scatter plots with regression lines and BKMR (Bayesian Kernel Machine Regression) analysis; Table S1: Linear or polynomial regression analysis.

## Abbreviations

The following abbreviations are used in this manuscript:

PD	Parkinson’s disease
MD	Mean diffusivity
AD	Axial diffusivity
RD	Radial diffusivity
FA	Fractional anisotropy
SN	Substantia nigra
CN	Caudate nucleus
PUT	Putamen
GP	Globus pallidus
DN	Dentate nucleus
RN	Red nucleus
BG	Basal ganglia
Al	Aluminum
Cu	Copper
Fe	Iron
Mn	Manganese
Pb	Lead
V	Vanadium
YrsW	Welding years
T1W	T1-weighted
UPDRS-III	Unified PD Rating Scale-motor-III
MANCOVA	Multivariate analysis of covariance
BKMR	Bayesian kernel machine regression
FWER	Familywise error rate
