# Supplementary material for: Subcortical Microstructural Changes Associated with Multiple Metal Exposure
Source: Toxics. 2026 Oct 15;14(10):911. doi: 10.3390/toxics14100911 (PMC13709804; doi:10.3390/toxics14100911)
Supplement: Supplementary file 1 [file toxics-14-00911-s001.zip › Supplementary Figure S1.pdf]

# Supplementary Figure S1

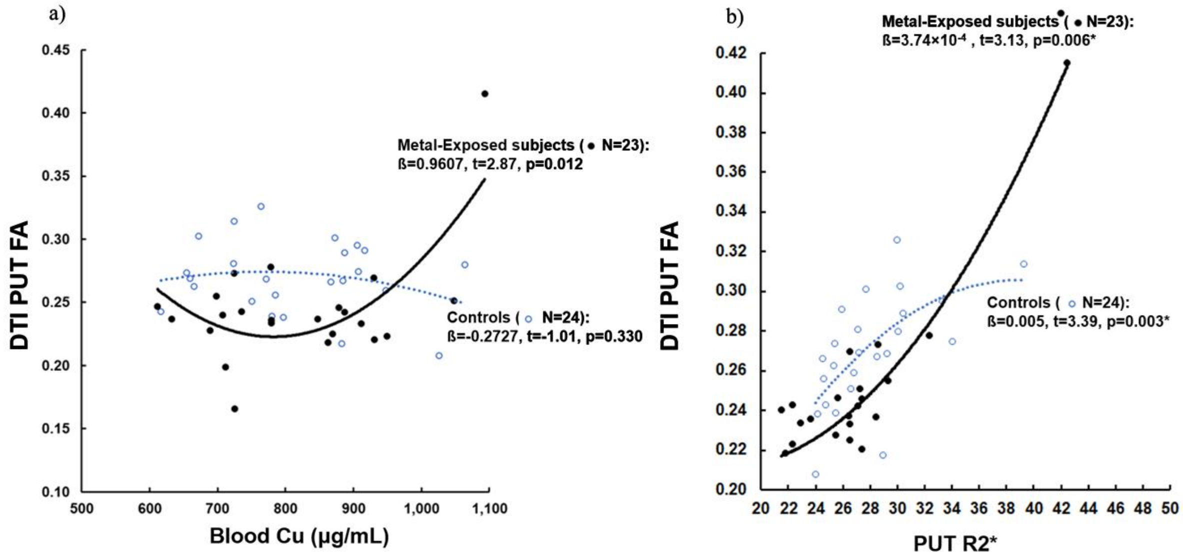


Scatter plots with regression lines showing diffusion tensor imaging (DTI) fractional anisotropy (FA) values in putamen (PUT) (DTI PUT FA; y-axis) vs. a) whole blood copper (Cu) levels (Blood Cu; x-axis), MRI R2* metrics in b) putamen (PUT; x-axis) for both metal-exposed subjects and controls. * indicates significant result at *FWER*=0.05.

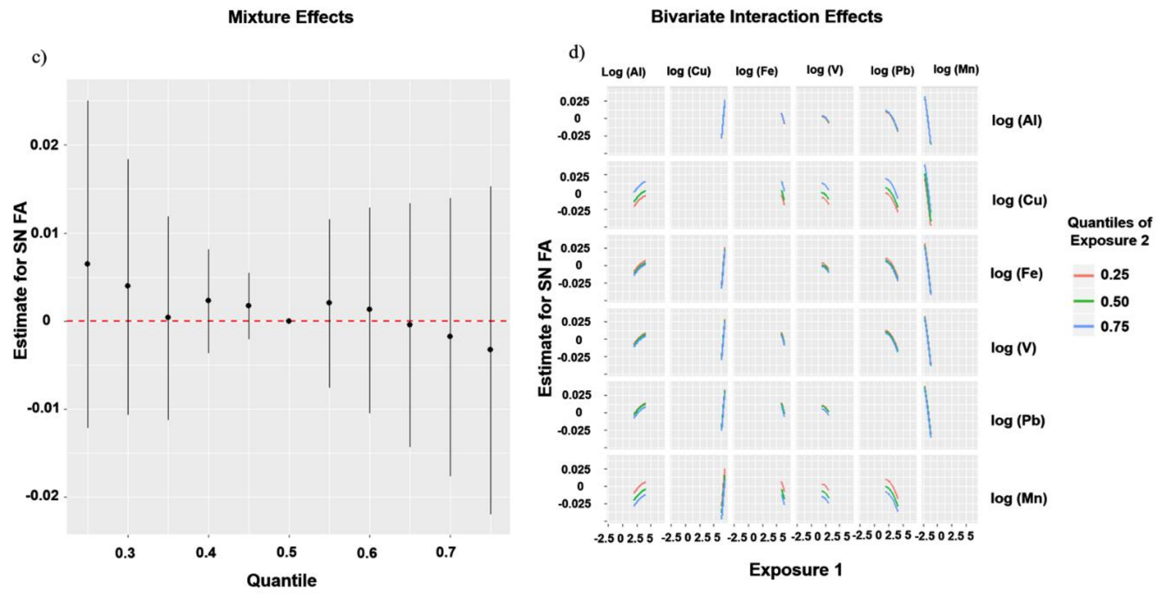


BKMR (Bayesian Kernel Machine Regression) analysis results showing the effects of c) overall metal mixture at various quantiles on fractional anisotropy (FA) values in substantia nigra (SN), as well as d) a bivariate exposure-response function of a single exposure variable (exposure 1) on SN FA values when the second exposure variable (exposure 2) fixed at qualities of 0.25, 0.50, and 0.75 and the remaining exposure variables fixed to the median.
