# Supplementary material for: Subcortical Microstructural Changes Associated with Multiple Metal Exposure
Source: Toxics. 2026 Oct 15;14(10):911. doi: 10.3390/toxics14100911 (PMC13709804; doi:10.3390/toxics14100911)
Supplement: Supplementary file 1 [file toxics-14-00911-s001.zip › Supplementary_Table S1.pdf]

Table S1. Linear or polynomial regression analysis evaluating the effects of exposure measures on MRI metrics for metal-exposed subjects with adjustments of age and smoking status.

Outcome variables	*Selected Predictors*	*Parameter estimates (ß)*	*t-value*	*p-value*
	Metal-exposed subjects (N=23)			
a. R1 metrics				
Linear				
GP	V	0.196	2.66	0.019
TH		0.060	2.48	0.027
	Mn	0.039	2.32	0.036
Polynomial				
GP	Pb	0.136	5.08	<0.001*
SN		0.103	8.55	<0.001*
b. T1W metrics				
Linear				
CN	Fe	-149.000	-2.75	0.016*
	V	114.237	3.85	0.002*
PUT	Fe	-171.333	-3.10	0.008*
	Mn	48.395	2.31	0.037
	V	138.486	4.56	<0.001*
GP	Fe	-167.360	-2.61	0.021*
	Mn	57.473	2.37	0.033
	V	161.363	4.60	<0.001*
DN	Fe	-167.034	-2.63	0.020
	V	136.890	3.95	0.002*
RN	Fe	-161.079	-2.70	0.017*
	V	117.567	3.61	0.003*
SN	Fe	-164.936	-2.84	0.013*
	Mn	49.645	2.27	0.040
	V	133.215	4.20	<0.001*
TH	Fe	-152.318	-3.01	0.009*
	Mn	44.500	2.33	0.035
	V	117.236	4.24	<0.001*
c. R2* metrics				
Linear				
PUT	Cu	24.992	2.70	0.017*
GP		18.448	2.33	0.036
RN		18.580	2.62	0.020
SN		17.285	2.71	0.017*
TH		7.728	2.32	0.036
d. Diffusion metrics				
Linear				
SN FA	YrsW	-0.0021	-2.54	0.020*
	Cu	0.1612	2.83	0.013*

	V	-0.0943	-2.82	0.014*
Polynomial				
PUT FA	Cu	0.901	2.47	0.028*
	PUT R2*	3.80×10^{-4}	3.16	0.006*
GP AD	GP T1W	0.15×10^{-5}	2.46	0.024*
MD		0.15×10^{-5}	2.43	0.026*
TH FA	TH R1	-29.608	-4.09	<0.001*
MD		49.588	3.31	0.004*
RD		59.227	3.97	0.001*

Data represents parameter estimates (ß) and corresponding *t*- and *p*-values of a-c) exposure measures (lifetime welding years and blood metals) to predict MRI metrics to estimate brain metal accumulation (T1 and R2* metrics) and d) metal exposure measures (lifetime welding years, blood metals, and MRI T1 and R2* metrics) to predict diffusion metrics. The analysis was adjusted for age and smoking status. * indicates significant results after Stepdown Bonferroni multiple comparison correction of $p<0.05$.
